# LTOT Patients’ Experience of a Portable Oxygen Unit and Health-Related Quality of Life—A Cross-Sectional Study

**DOI:** 10.3390/healthcare8020182

**Published:** 2020-06-23

**Authors:** Sebastian Möller, Bodil Ivarsson, Lars-Åke Nordström, Anders Johansson

**Affiliations:** 1 Department of Information Technology and Biomedical Engineering, Region Skåne, Lasarettsgatan 37, 221 85 Lund, Sweden; Lars.A.Nordstrom@skane.se; 2 Department of Clinical Sciences, Lund University, 221 00 Lund, Sweden; anders.johansson@med.lu.se; 3 Office of Medical Service, Region Skåne, 205 25 Malmö, Sweden; bodil.ivarsson@med.lu.se; 4 Department of Cardiothoracic Surgery, Lund University, 221 00 Lund, Sweden

**Keywords:** LTOT, Medtech20, EQ-5D, HRQoL, quality of life, oxygen supplementation, patient experience, medical device

## Abstract

*Background:* The purpose of the present study was to elucidate the experiences of long-term oxygen therapy (LTOT) patients with a portable oxygen unit and to describe the patients’ self-assessed health-related quality of life (HRQoL). *Methods:* The study employed a prospective cross-sectional design. Data collection entailed two questionnaires, namely the MedTech20 (patients’ experience of the medical device in four areas) and EQ-5D (HRQoL). The informants consisted of patients (*n* = 148) treated with such a medical device and that were registered in Skåne University Hospital’s database, Medusa. *Results:* In the domain *Sense of security* the informant felt *the equipment reliable and safe to use* and expressed *a sense of control for the user*. Regarding *Social participation*, the responses did not indicate the device to *facilitate leisure activities*, *movement outside the homes*, *traveling* or *everyday tasks* to a larger extent. The respondents did express a reduced sense of *compromised integrity*, with a minor effect on *Intimacy*. With regards to *Convenience*, the responses indicated the product to provide *Adaptability to personal needs*. Overall, a strongly affected HRQoL (*Your current health condition,* EQ-VAS Md = 50 (IQR 36–70)) with strong correlation with EQ-5D was seen. *Conclusions:* Informants experienced the portable oxygen unit as reliable and safe to use while giving a sense of control over the disease itself. A minor impact on social participation was reported, except for a reduced sense of compromised integrity. The patients also reported a strongly reduced HRQoL.

## 1. Introduction

Chronic respiratory failure (CRF) is commonly found as a late-stage feature of a number of chronic lung diseases, such as chronic obstructive pulmonary disease (COPD), pulmonary fibrosis, and lung cancer, but is also seen in a variety of cardiac and neuromuscular conditions. Chronic respiratory failure is characterized by a chronic inability to become saturated with O_2_, leading to severe dyspnea or breathlessness, in particular, during exercise [1]. Patients therefore experience a strongly reduced overall health-related quality of life (HRQoL) that in the case of COPD is even correlated with disease severity [2].

Long-term oxygen therapy (LTOT) for at least 15 h/day has been a common therapeutic approach in CRF for many years, although only COPD patients appear to benefit in terms of long-term survival [1]. In addition, patients that often rely on stationary equipment or heavy tubes appear to struggle to comply with use of LTOT for 15 h/day and indeed suffer from a reduced quality of life. Patients in a study in the Netherlands reported, apart from side-effects directly related to the use of the devices, complaints such as “restricted autonomy” and “feeling ashamed” as reasons for their non-compliance [3].

In recent years, more portable devices such as liquid O_2_ containers and, in particular, small battery-driven oxygen concentrators have been developed to overcome these problems. These devices have been shown to provide oxygen supplementation comparable to the heavier tubes, improving performance in the 6 min walking test as well as saturation and breathlessness compared with patients breathing air [4].

However, little is known about how patients experience the use of these devices in support of the development of a person-centered care perspective [5]. A few studies have taken the patient’s perspective, usually using text analysis of structured interviews [6]. While classical patient-reported instruments, such as the generic EuroQol 5-dimension questionnaire (EQ-5D) [7], have proven their value in assessing patients’ QoL in respiratory disease [2], they do not capture important aspects such as patients’ experience of the use of medical devices.

Recently, a new tool has been developed that assesses patients’ experiences associated with medical devices, namely the MedTech20 questionnaire [8]. MedTech20 is a generic questionnaire, and offers the possibility to elucidate the patient-perceived experience for different products and product categories in a manner suitable for health economic assessments. Both the EQ-5D and MedTech20 have the option to express health-related QoL as an aggregated index value, a QoL value that can be used to balance survival and health status into quality-adjusted life years (QALYs), which allows devices or treatments to be assessed for cost effectiveness.

The purpose of the present study was to elucidate patients’ experience of a portable oxygen concentration unit and to describe their overall self-assessed HRQoL.

## 2. Materials and Methods

### 2.1. Ethical Considerations

Ethical approval was obtained from the Regional Ethical Review Board in Lund, Sweden (Dnr: 2018/360), and the project was reviewed, approved, and funded by the Office of Medical Service and the Department of Information Technology and Biomedical Engineering at Region Skåne, in southern Sweden. All participants gave written informed consent prior to study participation. The study was conducted in accordance with the guidelines of the Declaration of Helsinki regarding the rights and dignity of participants.

### 2.2. Design

The study was designed as a prospective cross-sectional study using a descriptive closed-question survey methodology employing two questionnaires: the novel MedTech20 (Nordic Health Economics) and the well-established EQ-5D (EuroQoL Group).

The MedTech20 survey [9] examines patients’ experiences of medical devices and consists of 20 different product attributes in four areas: sense of security, social participation, integrity, and convenience [8]. In addition, a proprietary MedTech20© Index can be calculated.

The EQ-5D questionnaire provides a descriptive profile (EQ-5D) and a single index value for health status (EQ VAS) [7]. The descriptive questionnaire, EQ-5D, consists of questions where the individual can classify his/her own health in five different dimensions, namely *mobility*, *self-care*, *usual activities*, *pain/discomfort*, and *anxiety/depression*, on a three-dimensional scale (no problems, some problems, or severe problems). The individual’s responses to these questions then form a health profile that represents a specific state of health.

The EQ VAS records the informants self-rated health where the endpoints are labeled *Best imaginable health state* and *Worst imaginable health state*. This statement is then used as a quantitative measure of the health outcome as judged by the individual respondent.

The study recruited adult patients (>18 years) who used a common portable oxygen unit (Inogen One) that was registered in the medical device database, Medusa, maintained at the Office of Medical Service, Skåne. Data were collected during the second half of the year 2018 with two reminders sent for optimal data collection.

### 2.3. Statistics

Collected responses from MedTech20 and EQ-5D (questions 1–5) were separately transformed to numeric values (MedTech20: 1–7; EQ-5D: 1–3). The patients’ estimated *Your current health condition* (EQ VAS) was analyzed with the statistical software SPSS^®^ version 23.0 (SPSS Inc., Chicago IL). The nature of above collected variables is regarded as qualitative experiences and were accordingly analyzed with non-parametric tests (Mann–Whitney U test). Patient age were analyzed using T-test and gender (proportions) was analyzed with Chi-square test, and all mentioned variables are reported as absolute and relative frequencies, where appropriate. In addition, using proprietary algorithms and based on fully anonymized responses, the provider of Medtech 20 (Nordic Health Economics) calculated a MedTech 20 index on group level.

## 3. Results

### 3.1. Study Population

Out of 297 patients originally identified in MEDUSA, 282 eligible patients received an offer to participate in the study. A total of 148 informants (56 male and 92 female, 62% female) that provided responses to the questionnaires were included in the final statistical analysis (53% rate of response). The informants in this group had a mean age of 74 ± 10 years, with no significant age differences between sexes (male 76 ± 8 versus female 73 ± 11 years, respectively, *p* = 0.137).

Two informants (1%) did not submit answers to the MedTech20 questionnaire and 10 informants (7%) did not submit answers to the EQ-5D questionnaire. Five individuals caused a total of 10 missing values (0.3%) in the MedTech20 questionnaire and 16 informants (11%) did not respond to the EQ VAS question (Likert scale).

### 3.2. MedTech20

**Figure 1:**
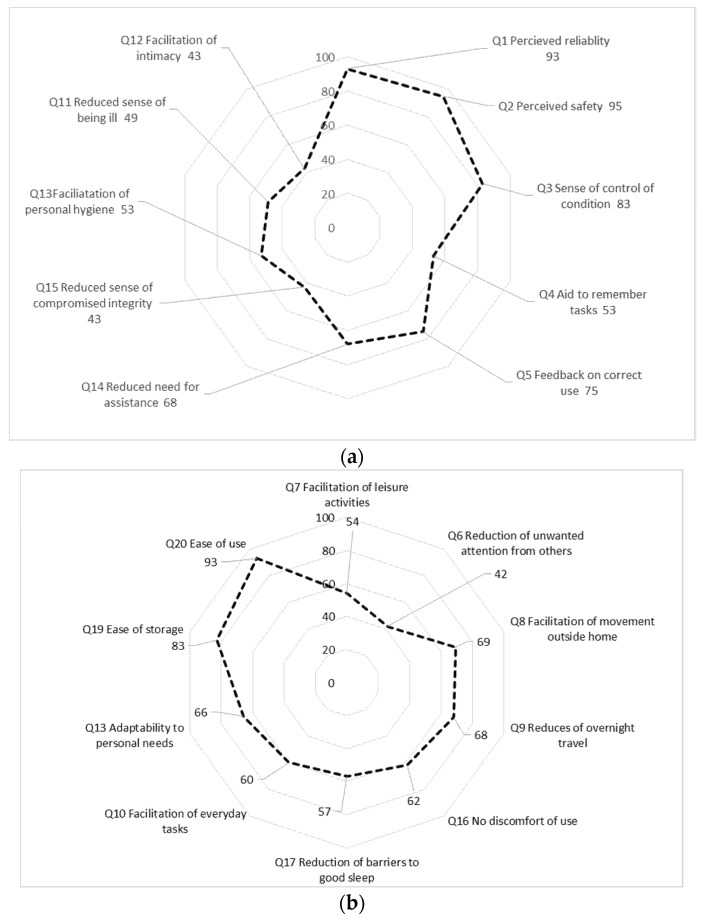
(**a**): Relative frequencies of responses of at least level 3 of 7 “agree”) to each question (Domain Sense of security question 1–5; Domain Integrity, Q 11 to 15); (**b**): Relative frequencies of responses of at least level 3 of 7 (“agree”) to each question (Domain Social participation Q7–9, 16; Domain Convenience, Q 10, 17–20.

Figure 1a,b summarize relative frequencies of responses to each question of at least level 3 (“agree”). For clarity, the response option “not relevant” in the questionnaire is not included in this figure but reported separately in Table 1.

In the domain *Sense of security*, a majority of the informants expressed that they perceived the equipment as *reliable* and *safe to use as well as a sense of control* (Figure 1a). However, only a minority of informants considered the equipment as an *aid to remember tasks* and, in fact, 40% of the informants thought this question was *Not relevant* to answer (Figure 1a).

In the domain of *Integrity*, the results indicate a *Reduced sense of compromised integrity*, but with minor effect on *Facilitation of closeness or intimacy* (Figure 1b). Notably, a majority of patients responded positively for questions on *Facilitation of movement outside home* (69%) or *Facilitation of overnight travelling* (68%). Around 24% of respondents considered question 14 (*Reduced need for assistance from others*, 24%) as irrelevant (Figure 1a).

In the domain of *Social participation*, the informants generally expressed lower confidence that the portable oxygen concentrators were helpful, with the exception of “reduction of unwanted attention” (Figure 1a). This domain also shows a large number of missing answers (between 3 and 42%, *n* = 5 questions) though the respondents respond that 3 of 5 questions are not relevant regarding this product (Figure 1b).

In the domain of *Convenience*, the respondents showed a high level of agreement to that ability of *Adaptability to personal needs* as well as to product *Ease of storage* and *Ease of use* (Figure 1b). However, even in this domain, some respondents felt that several questions were not relevant to respond to (10–17%, *n* = 3 questions, Figure 1b).

In the overall population, the proprietary MedTech20© Index was calculated as a mean of 0.554 (SD 0.191).

### 3.3. EQ-5D-Quality of Life

In all five dimensions of EQ-5D, the response by the patients indicate that they experienced a reduced HRQoL, generally with an overall greater percentage of women experiencing this type of problem (Table 2).

Notable is that the domain *Self-care* (some problems washing and dressing or unable to wash/dress oneself) seemed to affect health-related QoL (as measured as EQVAS values) most (correlation coefficient *r* −0.504, *p* = 0.001), while *Pain/discomfort* (moderate to extreme pain or discomfort) exhibited the weakest correlation (correlation coefficient *r* −0.193, *p* = 0.034, Table 3).

Patients assessed their overall health status (as EQ VAS), to 50 (IQR 36–70) on a 0–100 Likert scale. No differences in EQ VAS between sexes were observed (*p* = 0.624), nor did EQ VAS correlate with age in this population (Spearman’s Rho 0.29, *p* = 0.752).

## 4. Discussion

There is increasing demand for the evaluation of medical devices with respect to their usability as well as their intended effect. While a number of well-established instruments, both generic and disease-specific, exist for the (self-)assessment of HRQoL, such as EQ-5D [2], the lack of similar instruments for the assessment of the patient’s experience of the use of medical devices has limited our knowledge of this important aspect. Recently, a generic instrument, MedTech 20, has been developed [8], but to date, the experience with this instrument is rather limited.

In the southern region of Sweden (Region Skåne, approx. 1.3 million inhabitants), portable oxygen-concentrating devices have, today, almost completely replaced the use of pressurized containers for portable LTOT. While newer devices are comparably effective [4] with clear benefits from a cost and logistics perspective, less is known about how patients experience these devices in their daily use. The purpose of the present study was thus to elucidate patient’s experiences of the use of a portable oxygen unit (MedTech 20) as well as to describe the patients’ self-assessment of their HRQoL by the EQ-5D questionnaire.

Based on a regional inventory of medical devices, MEDUSA, maintained at the regional Office of medical Services, the population of our study consisted of patient’s using a portable oxygen-concentrating device for the treatment of a variety of medical conditions. The distribution of age, sex, EQ VAS resembles data from the national Swedish Oxygen register [10] and therefore suggest that our population is indeed representative for this group of patients in Sweden.

A previous questionnaire-survey study using the Swedish version of EQ-5D and EQ VAS was conducted in Sweden [11]. The more than 25,000 informants were recruited from the normal population and self-assessed their health on the EQ VAS scale to a median value of 80 (IQR 60–90). No differences between men and women were seen. This value was shown to decrease with age, with a median value of approximately 65 in the age group of 80–84 years, suggesting an expected median value of just over 70 in the age group present in our study. We observed in our study an EQ VAS median value of 50 (IQR 36–70), indicating that the patients in our study experienced a strongly impaired HRQoL compared to a normal population, as indeed would be expected from the burden of the diseases they suffer from. In the Swedvox register, a value of just under 50 was reported [10], again supporting a more general transferability of our results.

Our study is one of the first examples of the use of MedTech 20, a novel questionnaire for the assessment of patient’s experience of medical devices. We chose to use MedTech 20 in a group of patients that, on a background of different conditions, share the problem of suboptimal oxygen saturation, severely impairing their HRQoL. Independent of the underlying diagnosis, LTOT is thus a commonly recommended treatment option to relieve symptoms and improve HRQoL in these patients. Therefore, this population appears suitable to explore the usefulness of a generic instrument to study patient’s experiences.

To our knowledge, only a few studies have explored how patients experience the use of their medical devices [6]. One study in a comparable Swedish patient group used a qualitative approach based on text analysis of interviews [12]. Interestingly, the authors studied patients that were provided with LTOT at home using stationary equipment, and many of the complaints they voiced revolved around spaces such as “restriction to time and room” with statements expressing “The tube is an obstruction everywhere”, “Haven’t got the strength to take the apparatus with me”, or “Can’t take the car and be away over the weekend”. These statements stand in contrast to some of the areas in MedTech 20, where the patients expressed positive responses on questions such as *Facilitation of movement outside home* or *Facilitation of overnight travelling*. This difference may be attributed to the inherent mobile properties of the devices used in our study.

In our study, the MedTech20© Index was calculated as a mean of 0.554 (SD 0.191). While this index value is intended as a representation of the overall benefit patients experience with a medical device in their community, it is difficult to interpret based on only one study using MedTech 20. Future studies will generate richer material.

### Limitations

A number of circumstances may have affected the results of this study. Although our population appears representative of the larger Swedish LTOT patient community, informants with poorer health status than average as well as other confounding factors may be hidden in the composition of our study population with respect to their diagnosis and co-morbidities. These may have also contributed to the observed questionnaire dropout frequencies, and may thus have resulted in too high HRQoL values. However, low return rates are a well-known issue of postal questionnaire surveys [13] and, thus, not specific for this study. In the proportion of informants that did not respond to our questionnaire, there could have been a variation between sexes, affecting the calculated comparisons between the groups. Other possible circumstances that may have impacted on the result is that this study did not divide the material further into subgroups but presented descriptive data and analyses based on the total study population. Subgroups affecting responses to EQ-5D may include, e.g., education levels, different birth countries, or previous occupations.

## 5. Conclusions

The respondents in our study report that they suffer from reduced HRQoL and experience portable oxygen concentrators as reliable and safe to use. The devices seem to give their users a sense of control over their disease as well as a degree of freedom to participate in activities outside their home. Increased awareness of this aspect may help health professionals and healthcare managers to provide education and support to LTOT patients and their families. Presumably, since the MedTech20 questionnaire is a generic tool, we found a high frequency (up to 42%) of missing or “not applicable” responses for a number of questions, hampering their interpretation. Further studies will expand the experience with MedTech 20 and allow for deeper analysis of responses on different devices.

## Figures and Tables

**Table 1:** Frequencies of response option “Not relevant” in the MedTech20 survey, presented as absolute and relative frequencies and distribution by sex per question.

Frequencies Informant	Question 1	Question 2	Question 3	Question 4	Question 5
Total absolute (*n*)	0	0	1	57	8
Absolute male/female (*n*)	0/0	0/0	1/0	21/36	3/5
Total relative (%)	0	0	0	40	6
	Question 6	Question 7	Question 8	Question 9	Question 10
Total absolute (*n*)	7	61	43	5	51
Absolute male/female (*n*)	2/5	23/38	14/29	2/3	16/35
Total relative (%)	5	42	30	3	35
	Question 11	Question 12	Question 13	Question 14	Question 15
Total absolute (*n*)	17	20	5	35	1
Absolute male/female (*n*)	6/11	9/11	2/3	15/20	0/1
Total relative (%)	12	14	3	24	0
	Question 16	Question 17	Question 18	Question 19	Question 20
Total absolute (*n*)	24	20	15	1	0
Absolute male/female (*n*)	11/13	6/14	6/9	0/1	0/0
Total relative (%)	17	14	10	0	0

**Table 2:** Responses to EQ-5D. The descriptive system comprises 5 dimensions of standardized measures. Each dimension has 3 levels; no problems, some problems & severe problems. Results are described as absolute and relative frequencies (*n*/%) together with a statistical *p*-value between sexes. Analysis between sexes within EQ-5D, Pearson’s chi-squared test and between sexes within EQ VAS, T-test, respectively.

	Total	Men	Women	*p*-Value
Mobility *n* (%)				
No problems	31 (20.9)	15 (26.8)	16 (17.4)	
Some problems	103 (69.6)	38 (67.9)	65 (70.7)	0.311
Severe problems	2 (1.4)	1 (1.8)	1 (1.1)	
Self-care *n* (%)				
No problems	79 (53.4)	19 (58.9)	46 (50.0)	
Some problems	54 (36.5)	19 (33.9)	35 (38.0)	0.489
Severe problems	5 (3.4)	2 (3.6)	3 (3.3)	
Usual activities *n* (%)				
No problems	35 (23.6)	17 (30.4)	18 (19.6)	
Some problems	75 (50.7)	30 (53.6)	45 (48.9)	0.620
Severe problems	28 (18.9)	7 (12.5)	21 (22.8)	
Pain/discomfort *n* (%)				
No problems	29 (19.6)	15 (26.8)	14 (15.2)	
Some problems	90 (60.8)	34 (60.7)	56 (60.9)	0.880
Severe problems	18 (12.2)	5 (8.9)	13 (14.1)	
Anxiety/discomfort *n* (%)				
No problems	47 (31.8)	21 (37.5)	26 (28.3)	
Some problems	86 (58.1)	31 (55.4)	55 (59.8)	0.377
Severe problems	5 (3.4)	2 (3.6)	3 (3.3)	
EQ VAS Self-rated health				
Md (IQR)	50 (36–70)	50 (38.5–70)	50 (35–70)	0.624
Min-max	10–100	15–95	10–100	
EQ-5D index total population				
	0.620			

**Table 3:** Correlation values between Health-related EQ VAS and EQ-5D values, within the five domains Mobility, Self-Care, Usual Activities, Pain/Discomfort and Anxiety/Depression. Spearman’s rho, correlation coefficient (*r*).

	Correlation Coefficient (*r*)	*p*-Value
Self-Care	−0.504	0.001
Mobility	−0.463	0.001
Usual Activities	−0.335	0.001
Anxiety/Depression	−0.316	0.001
Pain/Discomfort	−0.193	0.034
